# Craniosynostosis, Scheuermann's disease, and intellectual disability resembling Shprintzen–Goldberg syndrome: a report on a family over 4 generations: Case report

**DOI:** 10.1097/MD.0000000000006199

**Published:** 2017-03-24

**Authors:** Ali Al Kaissi, Zahra Marrakchi, Nabil M. Nassib, Jochen Hofstaetter, Franz Grill, Rudolf Ganger, Susanne Gerit Kircher

**Affiliations:** a Ludwig Boltzmann Institute of Osteology, Hanusch Hospital of WGKK and AUVA Trauma Centre Meidling, First Medical Department, Hanusch Hospital; b Orthopedic Hospital of Speising, Pediatric Department, Vienna, Austria; c Department of Neonatology, Charles Nicolle Hospital, Tunisia; d Department of Pediatric Orthopedic Surgery, Children Hospital of Tunis, Tunisia.; e Institute of Medical Chemistry, Medical University of Vienna, Austria.

**Keywords:** array-CGH-analysis, case report, craniosynostosis, CT scan, intellectual disability, Scheuermann's disease, Shprintzen–Goldberg syndrome

## Abstract

**Rationale:**

Craniosynostosis is a disorder characterized by premature fusion of cranial sutures with subsequent development of abnormal craniofacial contour associated with variable skeletal and extra-skeletal abnormalities. In this family syndromic type of craniosynostosis was recognized and the etiology behind diverse forms of deformities have been diagnosed.

**Patient concerns:**

The negative impact of the disorder on the child and his family is enormous. Particularly when the diagnosis is late and little can be done. Though counselling the family through discussing the whole picture of the disorder might lessens their concern.

**Diagnoses:**

Diagnosis is the corner stone of management. In this paper we aimed to sensitize pediatricians, physicians, and orthopedic surgeons concerning the necessity to recognize syndromic associations early on.

**Interventions:**

Patients with syndromic craniosynostosis are usually associated with a complexity of malformation complex. Craniofacial surgery can be of remarkable help if the diagnosis is made early. It requires a series of corrections to avoid intellectual disability and other neurological deficits.

The timing of interventions is strongly correlated on the timing of diagnosis.

**Outcomes:**

The earliest the diagnoses, the much better the outcomes are. And consequently avert the psychological and the financial cost on the patient and his family.

**Lessons:**

The golden principle of medicine should prevail in all medical disciplines, which states: The more you see, the more you know and conversely the more you know is the more you see.

## 1 Introduction

The Shprintzen–Goldberg syndrome (SGS) shares many features with the Marfan syndrome, such as long arms, legs, and arachnodactyly. Craniosynostosis leads to abnormal craniofacial contour and results in a long narrow head, widely spaced eyes, high vault palatine, and low set ears. In addition to intellectual disability, congenital heart defects and muscular hypotonia in early infancy can be observed.^[1]^ The Lujan–Fryns syndrome is a recessive X-linked condition characterized by mental retardation, marfanoid habitus, and facial dysmorphisms.^[2,3]^

Scheuermann identified the radiographic characteristics of a specific type of angular kyphosis with anterior wedging of the vertebral bodies and irregularities of the vertebral apophyses.^[4]^ Scheuermann's deformity is the most common-cause of angular progressive structural thoracic or thoraco-lumbar kyphosis with associated back pain in adolescence. It has an incidence of 4% to 8% and no gender predominance. Radiographs are the standard imaging modality used to confirm the diagnosis of Scheuermann's disease. Classic signs include vertebral end plate irregularity, disk space narrowing, and anterior wedging of involved vertebral bodies.^[5]^

We describe a 12-year old boy, his mother, and other family subjects over 4 generations, in which all those who were affected had significant marfanoid habitus, arachnodactyly, and craniosynostosis associated with kyphosis. Spinal abnormality in the proband was diagnosed to be in connection with Scheuermann's disease. The degree of intellectual disability was sufficiently variable for those with a lesser degree of learning difficulty, to have children. All those who were affected had a characteristic facial appearance. Both the mother and her offspring had a craniosynostosis and proptosis. Both had high-pitched voices. The osteoporosis was marked. The mother and her child had very similar craniofacial contour.

## 2 Case reports

**Figure 1:**
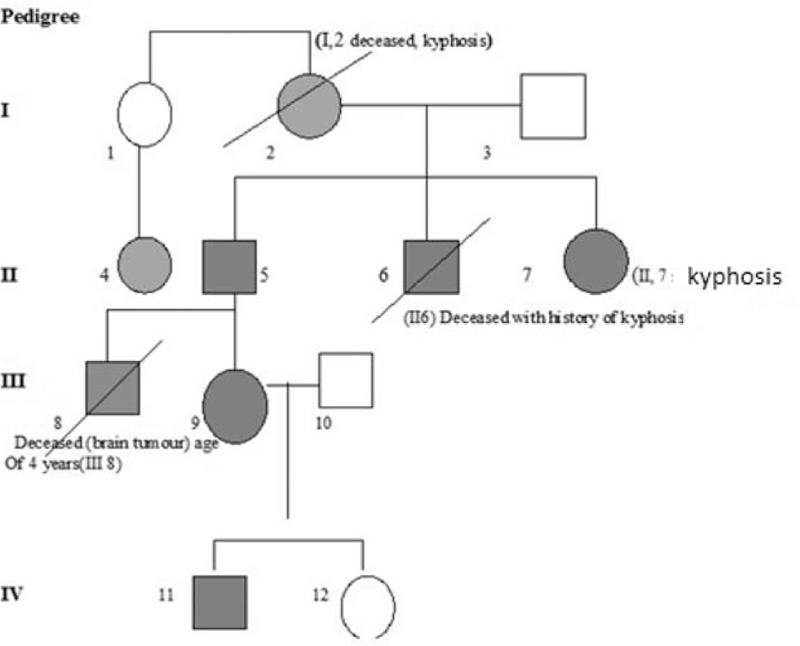
Pedigree of the family showing 4 generations with features observed in the 12-year old patient (IV, 11). All affected persons are in dark gray colored symbols, symptoms of the affected family members (see Table 1).

The proband, a 12-year old male child (IV, 11, see Fig. 1), was referred because of thoracic kyphosis, the offspring of a full-term gestation and was born to a 34-year-old woman and a 39-year-old unrelated man. The mother experienced an unexplained primary infertility for 3 years before giving birth to this male child. At birth, he was hypotonic. The boy sat without support at 11 months and he walked—despite frequent falls—at 23 months. His subsequent course of development in his first year of life was markedly retarded, particularly his motor development, the extreme ligamentous hyperlaxity, and profound muscular hypotonia, which raised concern of his pediatrician of the diagnosis of muscular dystrophy. Therefore, vigorous investigations were carried out at the institute of neurology. Serum creatin-kinase and plasma lactate were within normal range. Electromyography showed minimal myopathic changes, although muscle MRI imaging showed minimal, nonspecific changes. Muscle biopsy and muscle respiratory chain enzymes were normal, investigations to exclude selenoprotein-related myopathy (*SEPN1-gene*) and ryanodine receptor 1 (*RYR1-gene*)- related myopathy have been performed and showed no pathology. Chromosomal analysis was normal, metabolic disturbances were excluded, and screening for mucopolysaccharidoses was negative as well.

Other aspects of development, such as performance and schooling achievements were impaired. Hearing, vision, and neurological examinations were normal.

Because of his marfanoid-like habitus, ligamentous hyperlaxity and the arachnodactyly, the clinical diagnosis of Marfan syndrome has been established thereafter. Molecular genetic investigations showed no mutation in the *FBN1-gene*. We performed examination of the *TGFBR1- and TGFBR2-genes* in order to exclude the Loeys–Dietz syndrome. We excluded structural chromosomal aberrations and microdeletions or microduplication by array-CGH-analysis.

**Figure 2:**
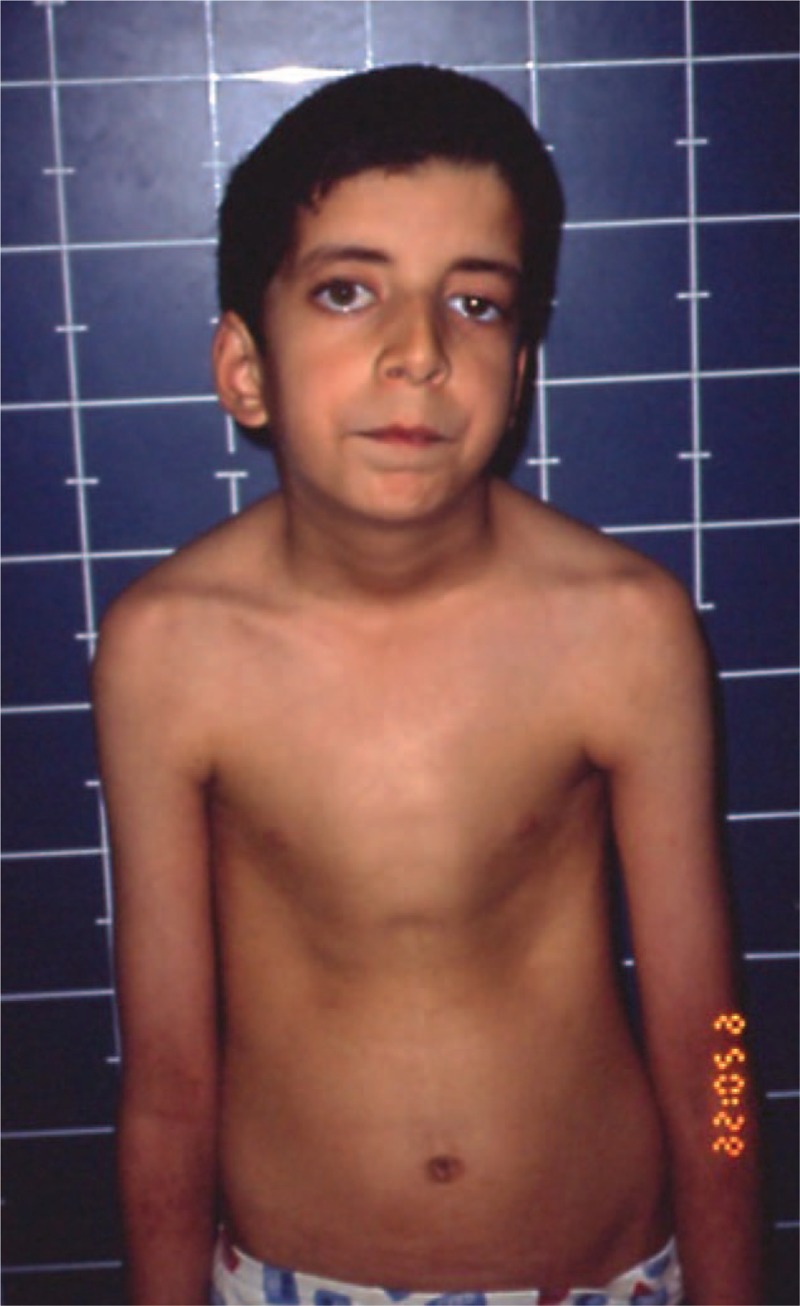
Clinical phenotype of the child showed plagiocephaly, facial asymmetry, a triangular face with a small chin, prominent low set ears, bilateral ptosis, epicanthic folds, hypertelorism, exophthalmos, maxillary and mandibular hypoplasia, long philtrum, and high palatine. His eyebrows were interrupted, there being almost a double layer and his palpebral fissures were long. Low-set large ears and he had a marked ligamentous hyperlaxity, arachnodactyly, and thoracic kyphosis. The trapezoids are profound because of thoracic kyphosis.

Clinical examination of the child revealed long limbs and thin body habitus (height 141 cm) associated with rigid thoracic kyphosis and arachnodactyly. Craniofacially, his head showed plagiocephaly (OFC: 53 cm), facial asymmetry, a triangular face with a small chin, prominent low set ears, bilateral ptosis, epicanthic folds, hypertelorism, exophthalmos, maxillary and mandibular hypoplasia, long philtrum, and a high vaulted palate. We noted a speech development test result close to the lower age specific limit. His eyebrows were interrupted, there being almost a double layer and his palpebral fissures were long. We noted low-set large ears. He had a marked ligamentous hyperlaxity, arachnodactyly, and thoracic kyphosis. The trapezoid muscles were profound because of thoracic kyphosis (Fig. 2). His stance, suggesting asymmetry in limb length, was a consequence of his kyphosis. He had a history of frequent fractures since early childhood; the same was recorded for his mother, who had a history of fractures twice in her life, the first at the age of 3 years and the second at 12 years.

**Figure 3:**
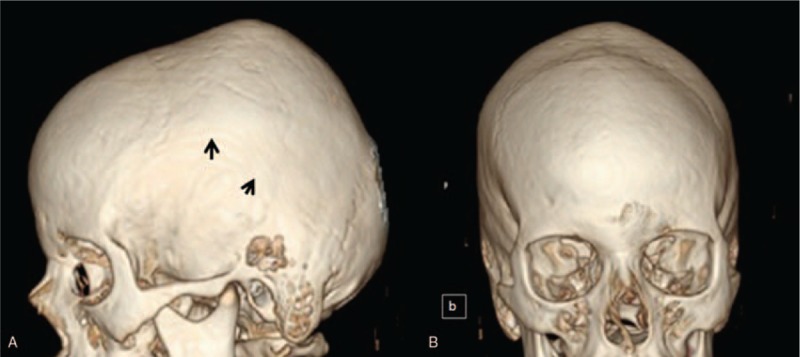
(A) 3D reconstruction CT of the proband skull showed severe expansion of the skull posterior to the coronal sutures because of mixed synostosis of the squamosal sutures (arrows) and the anterior part of the sagittal suture. The overall skull phenotype is somehow brachycephalic. (B) 3D reconstruction CT of the proband anterior view showed the massive bulging over the synostosed sagittal suture. CT = computed tomography.

CT scan of the proband's skull showed severe bulging over the sagittal suture due to synostosis. We noted bilateral synostosis of the squamosal sutures (arrows). The overall skull phenotype was brachycephalic (Fig. 3A). 3D reconstruction CT-scan of the proband's anterior view of the skull showed the massive bulging over the synostosed sagittal suture (Fig. 3B).

**Figure 4:**
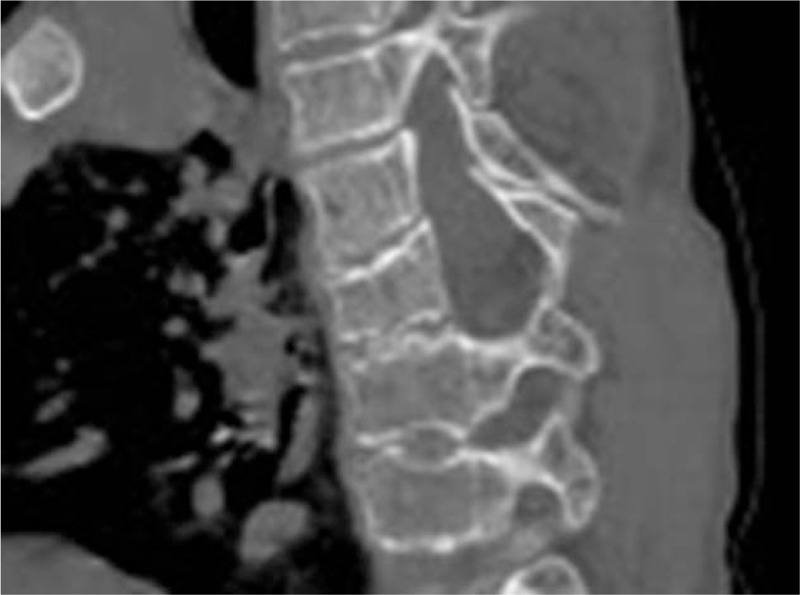
Coronal 3D-CT scan of the thoracic spine of the proband at age of 12-year showed wedging of 3 consecutive adjacent vertebral bodies in the apex of the kyphosis, irregular vertebral apophyseal lines combined with flattening and wedging, narrowing of the intervertebral disc spaces and variable presence of Schmorl's nodes, causing effectively the development of thoracic kyphosis (arrows). CT = computed tomography.

3D-CT scan of the proband's thoracic spine obtained at age of 12 years showed wedging of 3 consecutive adjacent vertebral bodies in the apex of the kyphosis, irregular vertebral spine apophyseal lines combined with flattening and wedging, narrowing of the intervertebral disc spaces and variable presence of Schmorl's nodes, causing thoracic kyphosis (arrows (Fig. 4).

**Figure 5:**
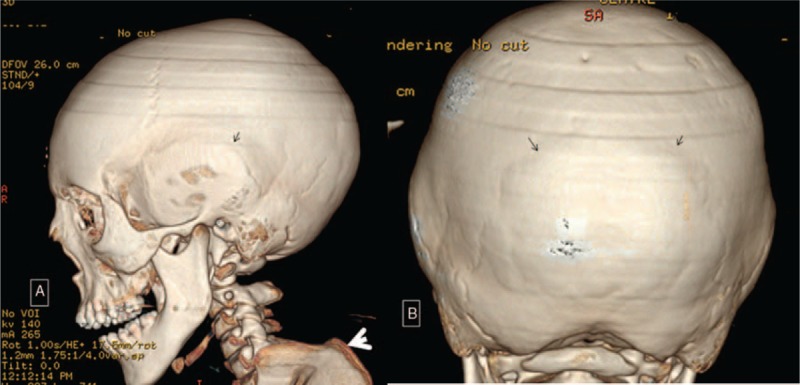
(A) 3D-CT scan of the mother skull showed bilateral bulging of the temporals. Synostosis of the sagittal suture with posterior bulging deformity akin to her son. Note the bulging of the synostosed squamosal suture (arrow) and the massive kyphosis (arrow head). Coronals are normal. (B) Mother 3D reconstruction CT scan showed diffuse synostosis of the lambdoids (arrows). Note the marked bossing of the occipital area. CT = computed tomography.

**Figure 6:**
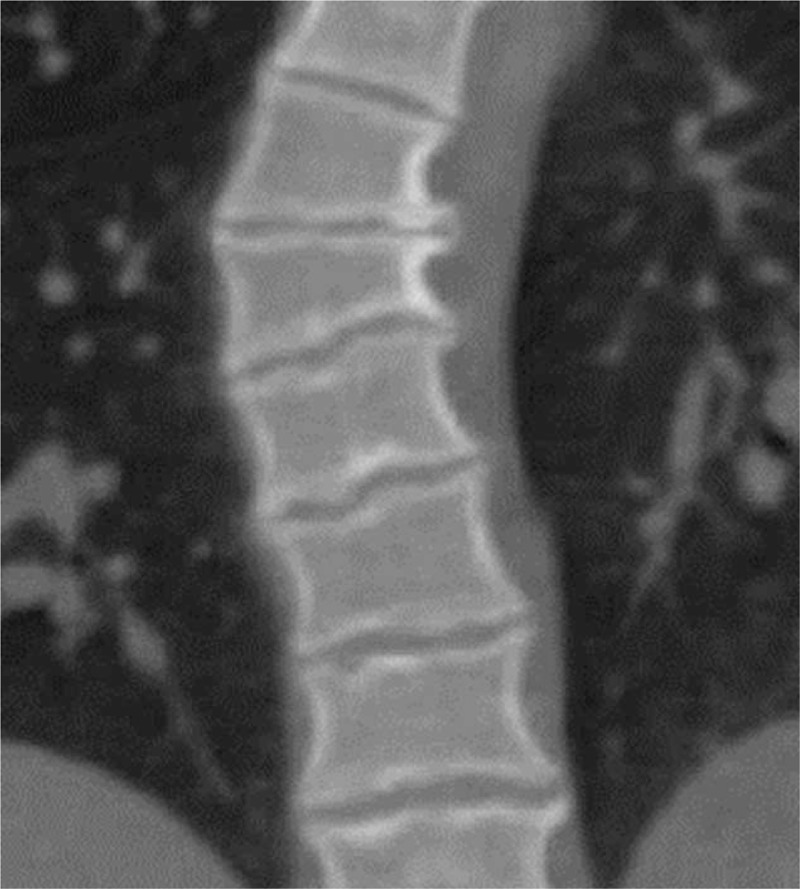
Sagittal 3D-CT scan of the spine of the mother showed trapezoid/wedge-shaped vertebral bodies at the T5–9 associated with irregularities of the vertebral apophyses, sclerosis, Schmorl node (arrow) with subsequent development of osteophytes around the vertebrae causing effectively the development of spinal osteochondritis. CT = computed tomography.

The proband's mother (pedigree III, 9) manifested short stature, but marfanoid-like habitus (body height 146 cm) and she had a history of poor school achievement. She developed thoracic kyphosis at the age of 11 years. Her face contour resembled the face of her son with a triangular face emphasized by the broadness of her forehead. Her mandible was pointed; she showed hypertelorism, exophthalmos, epicanthic folds, relatively long palpebral fissures, and similar eyebrows. Her trunk and neck were short, the feet and hands were broad, and there was pes planus and generalized ligamentous hyperlaxity. Skull radiographs showed craniosynostosis of the lambdoid and the sagittal sutures. We noted bilateral bulging of the temporal bones and synostosis of the sagittal suture with posterior bulging deformity. 3D reconstruction CT-scans showed bilateral synostostosis and bulging of the squamosal suture (arrows) and marked thoracic kyphosis (arrow head) (Fig. 5A). The 3D-CT scan reconstruction in his mother showed also synostosis of the lambdoid sutures (arrows) associated with profound bulging of the occipital area (Fig. 5B). We found posterior displacement of the ears in his mother. Sagittal 3D-CT scan of the spine obtained in the mother of the patient showed trapezoid and wedge shaped vertebral bodies from T5–9 associated with irregularities of the vertebral apophyses. We noted Schmorl node (arrow) with subsequent development of osteophytes around the vertebrae accompanied by osteochondritis (Fig. 6).

## 3 Discussion

Shprintzen and Goldberg described 2 unrelated males with craniostenosis, exophthalmos, maxillary and mandibular hypoplasia, prominent lateral palatine ridges, low-set soft ears, abdominal hernias, arachnodactyly, and camptodactyly.^[1]^ Milestones were delayed, and there was hypotonia and mental retardation. Surprisingly, a full skeletal survey was not reported on either of these cases, the differential diagnoses included skeletal dysplasias such as the Melnick–Needles syndrome. The phenotype of SGS syndrome is variable because it combines craniofacial, neurological, skeletal, cardiovascular, and other connective tissue abnormalities.^[6,7]^ Patients become increasingly dysmorphic with age with marked hypertelorism, shallow orbital ridges, marfanoid habitus, downslanting palpebral fissures, cleft palate, and marked micrognathia.^[8,9]^

Ades et al^[10]^ reported 4 girls who they suggested to have features of Shprintzen-Goldberg syndrome. However, radiographs did show many features of Melnick–Needles syndrome and some symptoms seem to overlap between the 2 conditions.^[6]^ Robinson et al^[11]^ reported 13 unrelated patients and 1 sib with SGS and compared their clinical findings with those of 23 previously reported individuals. They suggested that there is a characteristic facial appearance, with more than two-thirds of all individuals having hypertelorism, downslanting palpebral fissures, a high-arched palate, micrognathia, and apparently low-set and posteriorly rotated ears.

The marfanoid habitus, arachnodactyly, ligamentous hyperlaxity, kyphosis, and intellectual disability were the paramount features in our current family, besides to the craniosynostosis and Scheuermann's osteochondritis observed in the proband and his mother.

No instance could be found via searching the PubMed of the combination of a severe deforming Scheuermann's osteochondritis and intellectual disability and craniosynostosis inherited as an X-linked or autosomal dominant condition, that fitted the facial dysmorphism, kyphosis and Scheuermann's osteochondritis of the spine as seen in the proband and his mother.^[6–13]^ In this family, given the concordance of the 2 features in 5 out of the 8 affected members, it seems unlikely that the thoracic kyphosis is segregating independently from the craniosynostosis and the intellectual disability, and there is good evidence in this family of variable expression of intellectual disability.

Furthermore, looking at the clinical phenotype of the 3 generations shown in the family pedigree and (Table 1) showed the clinical presentation in other family subjects. Those affected are strikingly similar, giving the impression that the facial dysmorphism is part of the condition. Our current family is manifesting the main clinical features of SGS but with additional deformities which have been never reported before. The severity of retardation of the proband's family shows the main clinical features of SGS with additional deformities never reported before.

**Table 1:**
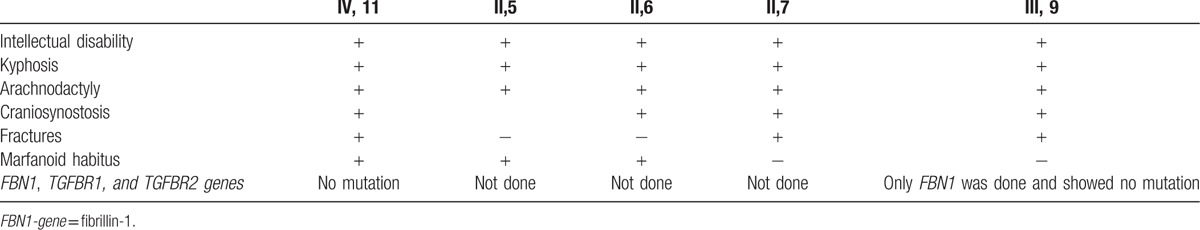
The variable skeletal features in 5 family subjects including the proband over 4 generations).

Lujan studied 4 males, the offsprings of 3 sisters, with mental retardation, a marfanoid habitus, a long narrow face, a large head, a high-arched palate, micrognathia, abnormal speech with a hypernasal voice, joint laxity, and borderline large testes.^[2,14]^ Mental retardation ranged from mild to severe. They noted significant behavioral problems, such as extreme shyness, autistic features, poor attention, or frank psychotic features. The family reported by Lujan has been found to have a mutation in the *MED12-gene.*^[14]^

Doyle et al^[15]^ performed whole-exome sequencing in a woman with Shprintzen–Goldberg syndrome and her unaffected parents and identified only 1 variant, a de novo heterozygous missense mutation in the *SKI*-gene. The mutation was not present in the unaffected parents or in SNP databases. Subsequent sequencing of *SKI*-gene in 11 more sporadic cases of SGS revealed heterozygous variants in 9 of the patients, including 7 missense mutations and a 9-bp deletion. The mutations were not found in SNP databases or in the unaffected parents in the 5 cases in which parental DNA was available. All 10 mutation-positive patients had skeletal muscle hypotonia and developmental delay; 8 of the 10 also had aortic root dilation, 1 had arterial tortuosity, and 2 had splenic artery aneurysms, which spontaneously ruptured in 1 patient.

Scheuermann's disease is characterized by wedging of 3 consecutive adjacent vertebral bodies in the apex of the kyphosis, irregular vertebral apophyseal lines combined with flattening and wedging, narrowing of the intervertebral disc spaces and variable presence of Schmorl's nodes. ^[4,5]^ The wedging was thought to be due to lack of development of the vertebral ring apophysis in preadolescents and it was thought that after ossification, at about 10 years of age, the deformity, a trapezoid wedging, could be seen radiographically. However, the vertebral apophysis does not actually contribute to the longitudinal growth (height) of the vertebral body and thus damage to it by vascular or mechanical mechanisms would not cause the characteristic wedging.^[16]^ Mechanical, vascular, hormonal, nutritional, traumatic, and metabolic causes have been proposed as the etiological background for spinal osteochondritis.^[17]^ Nielsen and Pilgaard^[18]^ described a similar pedigree that was also segregating for autosomal dominant anterior spinal fusion. Halal et al^[19]^ described an autosomal dominant pedigree of Scheuermann's osteochondritis of the spine.

The sutures generally fuse at the end of the second year of life. Virchow explained that fused sutures act as barriers to the brain's normal growth, forcing the brain to expand in abnormal directions and thus creating a visible skull deformity. He observed that, acting as the underlying and expanding matrix of the skull, the brain and its development and growth directly and strongly affect skull growth and shape. He noted that when a suture is synostosed, the rapid force of brain growth is restrained and altered because the brain cannot expand in the direction of the synostosed suture. The brain and the skull shapes are thus forced in dimensions perpendicular to the fused suture as a consequence of a compensatory growth along the adjacent open sutures. This theory is still commonly accepted today.^[20]^

Surgical correction of craniosynostosis has to be done early in the first year of life. The management of craniofacial syndromes includes correction of craniosynostosis between 3 and 6 months of age. With age, skull deformation increases in severity and permanency, increasing the number of procedures necessary, the extent of the surgery, and the risk of complications are to be considered.^[21]^

Potential intraoperative complications include massive blood loss and air embolism, also it is mandatory for monitoring the head circumference and checking for signs and symptoms of increased intracranial pressure.^[22]^ The surgical management of craniosynostosis involves the early excision of the synostosed suture while brain growth is still rapid.^[23]^ The aim is to correct both the progressive cosmetic deformity and to re-establish the normal intracranial fluid pressure dynamics.^[24]^ The necessity of early diagnosis and intervention allow for the growing of the developing brain and restoring the craniofacial growth patterns.^[25]^ Drake et al^[26]^ debated the usefulness of craniectomy in children with craniosynostosis. Recurrent synostosis and hyperostosis represent adverse outcomes. Hyperostosis may increase intracranial pressure and further restrict the growing of the brain.^[27]^ Panchal et al,^[28]^ performed a retrospective analysis on children who underwent strip craniectomy or subtotal calvarectomy at age less than or greater than 4 months. The authors compared mean cranial indices (defined as the ratio of maximal cranial width divided by maximal length multiplied by 100) which were measured by 3-dimensional CT scan before surgery and 1 year following treatment. They found that children who underwent strip craniectomy, even if performed before 4 months of age, did not achieve a normal cranial index. Those children who underwent subtotal calvarectomy, however, did achieve a cranial index that resided within the normal range if the surgery was performed before age 13 months. Interestingly, the authors found no difference in outcome between infants who underwent surgery at mean age 2.9 months versus those who underwent surgery at mean age 7.6 months.

## 4 Conclusion

We believe this sort of abnormalities encountered in this family is interesting, primarily from the cranial and the facial bones changes characterized by a triangular shaped face. The joints are hypermobile due to increased laxity of the ligaments and tendons. The muscular bulk in the family looks hypotonic and to a certain extent, in the mother and the proband it looks atrophic. The skin is thin and has a fine texture giving rise to early ageing. The history of fractures and the generalized osteoporosis can lead to the misdiagnosis of osteogenesis imperfecta, despite no history of blue sclera, no wormian bones and no further fractures in other family members over 3 generations could be observed. In conclusion, we report a condition, characterized by marfanoid habitus, craniosynostosis, arachnodactyly, thoracic kyphosis, Scheuermann's osteochondritis, and a history of fractures. Given the distinctive features of SGS this should represent a recognizable syndrome.
